# Protocol paper for healthy ageing for Indigenous communities in India and Sweden with focus on nutritious and culturally adequate food provision

**DOI:** 10.1186/s12889-025-24651-7

**Published:** 2025-11-06

**Authors:** Ildikó Asztalos Morell, Monalisha Sahu, Vidya Sagar, Anit Kujur, Dewesh Kumar, Prabir Kumar Chatterjee, Dhananjay Singh, Lena Maria Nilsson

**Affiliations:** 1 Department of Urban and Rural Development, SLU Swedish University for Agricultural Sciences, Uppsala, Sweden; 2 Department of Occupational Health, All India Institute of Hygiene and Public Health , 110 CR Avenue Kolkata, Kolkata, India; 3 Department of Community Medicine, RIMS, Bariatu, Ranchi, 834009 India; 4 Amader Haspatal, Bankura, India; 5 Sanmat, Ashoknagar, Jharkand India; 6 Department of Epidemiology and Global Health, Umeå University, Umeå, Sweden

**Keywords:** Indigenous, Care regime, Food provision, Culturally sensitive care, Food security and sovereignty

## Abstract

**Background and purpose of study:**

The elderly care regime for Indigenous peoples differs between India and Sweden. In India, the family cares for the elderly, while the responsibility in Sweden lies with the authorities. Food insecurity is the main problem in India, while lack of culturally adapted food is a problem in Sweden. In both cases, little knowledge exists on the importance of traditional food in Indigenous elderly care. By examining the use and significance of traditional food in elderly care for the Mal Paharia people in India and the Sami in Sweden, we focus on the following questions: What is the significance of nutritious and culturally adequate food? How are the elderly care regimes for food constituted? How can these regimes be improved using participatory methods? What policy recommendations can be created based on our study?. Our study focuses on the health, organization and welfare aspects of aging from an Indigenous perspective.

**Methods:**

A consistent perspective on this study is the decolonialized and liberating action research with Indigenous peoples (so-called PAR). The method is based on a triangulation and mixed-methods design and is made up of four different sub-studies: WP1 on Indigenous life stories about food; WP2 on quantitative surveys and nutrition index; WP3 on care regimes and WP4 on participatory implementation. The research groups in India and Sweden, which include both Indigenous and non-Indigenous people from each country, will collaborate through a consortium. Focus group interviews will be conducted both with the elderly (WP1) and with government representatives and recipients (WP3). The quantitative study (WP2) is based on already collected data material from more than 1000 elderly Sami in Sweden, the Sami Health on Equal Terms study and on data to be collected among 200 older Mal Paharia (two-stage clustering) in India. Interpreters will be offered for people who communicate best in their mother tongue. The implementation study (WP4) will be carried out with a bottom-up perspective, with a focus on food safety in India and food sovereignty in Sweden.

**Discussion:**

Our study focuses on the health, organization and welfare aspects of aging from an Indigenous perspective. Older Indigenous peoples have knowledge about nutritious diets that are important to utilize, both from a care perspective, and from a longer-term self-sufficiency perspective, where their knowledge can give us keys to a more sustainable, culturally richer and biotope-wise more well-adapted future food system.

## The purpose of the research

This research project aims to do a comparative collaborative study focusing on two Indigenous people: Sami in Sweden and Mal Paharia in India between 2022-2024:

To record the view of the elderly Indigenous and other concerned stakeholders on what is nutritious and culturally adequate food.

To explore the importance of culturally adequate nutritious food to enhance active ageing of Indigenous older adults.

To describe the comparative features of the food and care regimes within which the dietary needs of Indigenous older adults are met currently using both qualitative methods and quantitative surveys in different seasons.

To employ locally acceptable participatory methods to engage older adults in defining possibilities to achieve culturally and nutritiously sufficient food.

## Background

Issues of ageing in Indigenous populations have been underresearched [1, 2]. Active ageing goals globally have been crucial principles for ageing worldwide, which places desirable ageing on the pillars of good health, participation and safety [3]. Nutritious food is important for maintaining an active, healthy and secure life. Food is also central for cultural identity and wellbeing [4]. Based on this distinction, one can talk about food security (to have access to enough nutrition) and food sovereignty (to be able to have control over the type of food and resources of food we eat) [5, 6]. This latter is tightly connected to the overall capability of Indigenous groups to maintain their culture, traditional livelihoods and access to nutritious food [7].

Active ageing is tightly related to the forms of provision of care for older adults [OAs] [8, 9], how traditional forms of care transform due to socio-economic and cultural changes [10–12], and how local forms of provision are embedded into national systems [13].

The capabilities [14] of IOAs to gain recognition for culturally adequate dietary needs and to participate in decisions concerning the provision of care and food [15] is circumscribed both by being resource poor within their own communities, i.e. malnutrition rate is highest among OAs in scheduled tribes [16] and by being members of underprivileged groups within dominant care regimes [17] adding to jeopardy related to ageism. Therefore, rather than doing research **for** IOAs this study aims to work with IOAs using health promoting participatory empowerment methods [18, 19].

### Indigenous older adults in Sweden and India

The collaborative project is to be carried out in two specific Indigenous contexts where, beyond common features, such as colonial past, ongoing threats to Indigenous food systems, their conditions, and livelihoods as well as experienced deficiencies of food provision differ.

Sámi are the only Indigenous People of EU and their traditional homeland, called Sápmi, is situated in northernmost Norway, Sweden, Finland and the Kola Peninsula of Russia. Their traditional sustenance includes reindeer herding, fishing, hunting, gathering, and small-scale farming, tightly connected to traditional Sami diet. Most Sami belonging to reindeer herding communities RHC nomad between seasonal pastures. While historically Sami moved with their ageing adults, lately OAs choose sedentary life in municipal centra. Many “city Sámi” work in other employment sectors of society. Sámi traditional food consumption varies among different sub-groups [20]. In Sweden, Sámi enjoy minority rights. Among these, municipalities can apply for special SCGM status to provide culturally adequate service for Sami. This includes seldom traditional diet in care service [21]. Thus, concerns for OA Sami are mainly related to food sovereignty.

Scheduled Tribes (ST) form 8.6% of India’s population with a much lower life expectancy (63.9 years) than the population at large [22, 23]. More than half of the 3.9 million ST adults over 60 years are working for subsistence, which is higher than OAs generally [24]. After 2006, out of 705 STs 75 were classified as Particularly Vulnerable Tribal Groups PVTG. PVTGs have low literacy, live forest-based livelihood, on pre-agricultural level of subsistence economy, with a stagnant or declining population. Nine of them reside in the state of Jharkhand [25].

Mal Paharia, a PVTG predominantly located in Dumka district, has been described as having a symbiotic bond with the forest for bodily and cultural survival [26]. Traditionally, they practised shifting cultivation and their food plate mainly consisted of fruits and flowers from the forests [27, 28]. From early 19th century, outmigration for employment has been significant, leaving behind the older generation to fend for themselves [29, 30]. The majority of MP OA live in abject poverty, dependent on employment and government doles [public distribution system rice] struggling with a high degree of food insecurity and malnutrition.

### Care and health regimes and provision of food for older people in Sweden and India

The care regimes, i.e. how care responsibilities are divided between the family, state, municipalities, market and civil society [8] diverge. In Sweden OAs get pensions based on their livespan income, financed by the state (to a minimum level) and their former employers. Health and care are provided by municipalities and health care regions and financed by local and regional taxes. Civil society plays a complementary role [9]. Elderly seldom live with younger relatives [31].

Individualistic values prevail, where welfare institutions and rights make it possible for OAs to live independent of their children. Facing one of the most advanced ageing populations the increased demand for care has prompted an increasing responsibilisation of families for the care of OAs [8]. State provision of elderly care is strictly monitored and is provided for duties that the OA cannot carry out themselves. What is typically left unattended is such needs of elderly that are non-physical including to cater culture adequate service [32].

In India most OAs rely fully on the provision of care by kin [33] and some [34] argue, that the family care system is weakening due to increased mobilities, leaving elderly in families unattended. The Maintenance and Welfare of Parents and Senior Citizens Act of 2007 declares OAs’ right to take legal actions against adult children for provision. The act contains no assurances for childless adults, and is difficult to enforce, especially among Mal Paharias who live in nuclear families [27, 35].

State welfare provision to elderly is rudimentary [36]. Only 20% of old people have independent pensions, with a lower proportion among women. The social/pension security system ignores the vast differences between elderly (e.g. rural and urban), with a large group of very poor, falling outside of social security systems. The situation is worse among ST [37] leading to food insecurity.

The National Program for the Health Care of the elderly [38] addresses the health care access of the elderly. Little is known about the reach of such programs to Mal Paharia elderly. Few studies on other PVTGs [39] indicate a widespread household food insecurity [40].

Among Mal Paharia it is common that grown-up sons set up a new family leaving behind the elderly [28], in a situation of social alienation, economic vulnerability and violated health [41].

### Traditional food systems among Indigenous people and the food security of aged populations

Traditional Sámi food items such as reindeer meat and wild berries are shown superior in nutritious content compared to farmed/cultivated food items [42] and serve as a mineral and protein rich diet fit to the climatic conditions of long freezing periods [7]. Imported food has lower nutritious value having adverse impacts on the health of Indigenous populations [43]. Thus, decline in traditional food in the Arctic results both in loss of nutritiously favourable food resources and the violation of continued cultural practices [42]. Reindeer herding remains crucial for cultural identification and food consumption among Sámi [44], and has special meaning for elderly Sámi as food biography interviews among Sámi in Norway and Russia emphasise [45].

Meanwhile, new studies are needed to address how the changed diet of OAs in municipal care provision who are not served traditional food, impacts their health. Whether it results in negative health spirals both due to food of lesser nutritious quality, and the loss of connectivity to their own culture and identity.

Mal Paharias who are in regions with extensive natural resource exploitation leading to deforestation and the loss of ability to access traditional food from nature, has largely contributed to malnutrition of the population. Thus the main concerns for OAs in Mal Paharia in India have been food security. Mean Per Capita Income (MPCE) is the lowest among the OAs belonging to scheduled tribes [37]. Due to low incomes and decline of traditional food sources obtaining nutritious food (food security) has become a challenge for tribal communities in India.

The median intakes of all the nutrients by OA male and female STs was shown to be below the Recommended Dietary Intakes (RDI). Sinha stressed the need for improvement in the nutritional status of elderly people by disseminating low-cost sustainable agriculture technologies along with nutrition education. Older women suffer disproportionately from malnutrition in tribes [46]. Malnutrition can be seen as a key contributing factor to low life expectancy.

### Ageing in Indigenous communities and state and civil society interventions

Sweden is a multicultural society and Sami are the only Indigenous population within the EU and Sweden and enjoy special rights in Sweden: they are acknowledged as people (that brings their status in the level with Swedes in principle) and are also one of Sweden’s national minorities. The 27 municipalities which applied for the status *Sami cultural governance municipality* SCGM (förvaltningskommun) are offered special rights and services. The issues of multicultural ageing have been problematized from the perspective of OAs rights and access to culture sensitive care, however, primarily in relation to immigrant and Finish minorities [32, 47] with focus on demands concerning financing family care (cash for care), and issues like the provision of service in the mother tongue [17].

Thus, the care provision for Sami with special status is a blind spot. Among few studies on preferred health care among Sámi in the end of life (DöBra-project) highlighted food as an important element [1]. So far, there is no research focusing on the culture sensitive care provision for Sami in Sweden with regards to food sovereignty even though Sami organisations [21] have raised the need to address municipal food provision, which follows standardised governmental recommendations and is governed by economic regimes, where cost-effectivity is strictly observed. This enhances the use of economies of scale, producing standardised food Sami food for elderly falling outside the radar.

In India the special concerns for tribal groups are pursued by needs assessment schemes by the government and the civil societies. Tribal communities suffer from Multiple disadvantages [48]. Under the National Health Mission (NHM) [49] all tribal districts whose composite health index is below the State average have been identified as High Priority Districts (HPDs) and receive more resources per capita under the NHM. Even those not covered under HPDs receive higher per capita funding, relaxed norms, enhanced monitoring and focused supportive supervision, and encouraged to adopt innovative approaches to address their peculiar health challenges. ‘Time to care’ Sub Centres were initiated within 30 min of walk from habitation and relaxed norm for Mobile Medical Units (MMU) for tribal areas; An Umbrella Programme for Development of Tribes – Van Bandhu Kalyan Yojana [50] was broadly a process, aiming at overall development of tribal people with an outcome-based approach, while striking at the critical gaps in the sectors of Housing, Livelihood, Health & Sanitation, Drinking Water, Agriculture & Irrigation, Electricity, Education, Skill development, Sports & Games and Preservation of Cultural Heritage.

The Right to Food and People’s Health Movement (Jan Swasth Abhiyan) [51] has all India networks. Their Jharkhand branch is very active bringing to light systemic neglect of tribal communities. At a National Human Rights Commission hearing at the turn of the century a case of neglect of Sauria Paharia families was taken up by Right to Health activists of PHM. There are several NGOs active in the vicinity of the Mal Paharia group also, such as the Social Development Centre (Catholic) of Dumka Diocese, the Santal Paharia Seva Mandal (Gandhian) with headquarters at Deoghar and a clinic at Gopikandhar, Manavi working with tribal women in Dumka. Sanmat is also in the process of training tribal women workers (health ambassadors) in that block who will work among Santals and Mal Paharias.

Though there are so many programs surrounding the STs in India, the plight of elderly PVTGs like Mal Paharia is still overlooked. Welfare of the PVTG elderly population demands attention on par with the general population in promoting active ageing among them and adding years to their lives.

## Active ageing and missing voices: A capability theory of health

The World Health Organization’s (WHO) new policy paradigm of active ageing [3] emphasizes preventive strategies to promote the well-being of older adults (OAs). This framework extends beyond the prevention of disability and disease and the maintenance of cognitive and physical functions—it also prioritizes social engagement, participation, security, and the improvement of conditions necessary for good health, all aimed at enhancing the quality of life for OAs.

The active ageing paradigm has gained international traction, including in countries like India and Sweden [39]. However, it has also faced considerable criticism. Scholars have questioned how the concept is translated into practice, its sensitivity to the diverse social, cultural, and medical contexts of older adults, and the degree to which care recipients are involved in shaping relevant interventions [52].

It has rarely been considered the plight of marginalised tribal groups nor, not counting some exceptions [39], tried to quantify the overall well-being of IOAs. Comparative assessment of the index implies challenges e.g. as to how to value older ST adults’ high labour participation. While this is an outcome of poverty and necessity, in the framework of active aging it counts as sign for active ageing.

**Fig. 1:**
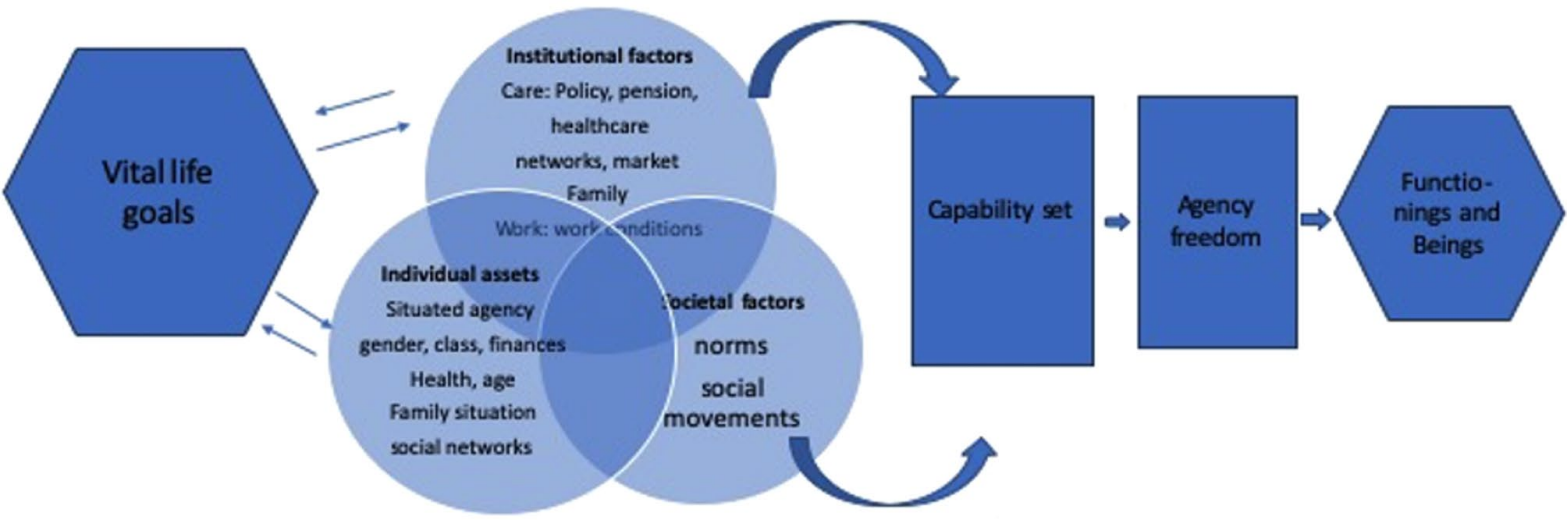
Capability approach

Instead of emphasis on active ageing, Nordenfelt proposed the Welfare Theory of Health (WTH), which defines health in terms of a person’s ability to achieve their individual vital life goals (VLGs) within specific spheres of life [53, 54]. Similarly, Sen’s Capability Approach [14] (see Fig. 1) is also grounded in the concept of VLGs, focusing on the “functionings” individuals are able to perform (what they can do) and the “achievements” they realize (the beings they can be) that they personally value [12]. However, Sen shifts the focus to individual freedom and agency, highlighting the gap between one’s capabilities and their ability to act on them—the so-called agency/capability gap.

Building on Sen’s framework, Hobson [55] and Robeyns [56] emphasize the critical role of social institutions and normative structures in shaping individuals’ capability sets. These structures can either enable or constrain people’s agency by influencing their opportunities to pursue and achieve outcomes they value.

Drawing on the Capability Approach as “an evaluative space to assess well-being and quality of life and the freedom to pursue it” [55], this study adopts a notion of agency that considers choices made within the constraints of existing “opportunity structures.” In applying this framework to older adults (OAs) in the context of food and health, the study examines how they pursue valued goals by converting entitlements into capabilities that contribute to a better life. These conversions are understood as responses shaped by the capability sets that OAs have accumulated over their lifetimes, which are, in turn, influenced by specific normative and institutional contexts.

Hobson’s [55] emphasis on the role of societal norms aligns with Spivak’s theory of the “subaltern” [57], particularly her critique of how the monolithic colonial construction of the “Third World woman” silences marginalized voices. Spivak’s work has significantly influenced postcolonial theory, especially debates around the politics of “giving voice” to minorities and socially marginalized groups and social recognition to the experiences and perceptions of postcolonial subjects [58]. A decolonial perspective [59, 60] is based on respect to Indigenous people’s knowhow and perspectives and appropriate it with the same status as “research”-based knowledge: research ***with*** Indigenous people and not for. Capability perspectives allow to highlight IOAs’ preferences and identify gaps between their vital life goals and “functionings” and how their capability sets, constrained by inequalities of resources, norms and institutional functionings explain this and by this can serve as ground for participatory capacity building.

Freire’s principles [61] for emancipatory participatory action research harmonize with Participatory Rural Appraisal and the Participatory Learning Action in India as introduced in Ranchi before Jharkhand got its status of a separate state [62, 63]. Freire [16] argues for the importance of engaging the resilience of vulnerable groups as the source for transformation: a participation-based empowerment and capacity building of vulnerable groups [18]. Capacity building assumes that those to be empowered reach “critical consciousness” about the inequalities, social injustice they would like to improve, to transform their feeling of helplessness to a mastery of their life. Meanwhile, institutional changes are required for long term transformation [18, 19].

Empowerment, “to increase control over, and to improve” human condition is as key element of health. To approve individuals’ functional (ability to access), interactive (use socially knowhow) and critical (assess critically) “health literacy” has been seen crucial to improve individual and community health [64, 65]. “Health literacy” to be critical to empowerment from a decolonial PAR perspective “must focus on the social determinants of health with an emphasis on the individuals’ and communities’ own perceptions of what health and quality of life is for them.” [66].

## Methods and design

The study design builds on methodological triangulation and mixed methods design and is constituted of four types of interconnected studies. The overall approach for this study is based on a decolonial Indigenous emancipatory PAR [60, 66] embark on a collaborative approach seeking bridges to Indigenous notions of health and those of codified science, without beforehand establishing a hierarchy of knowledge.

**Fig. 2:**
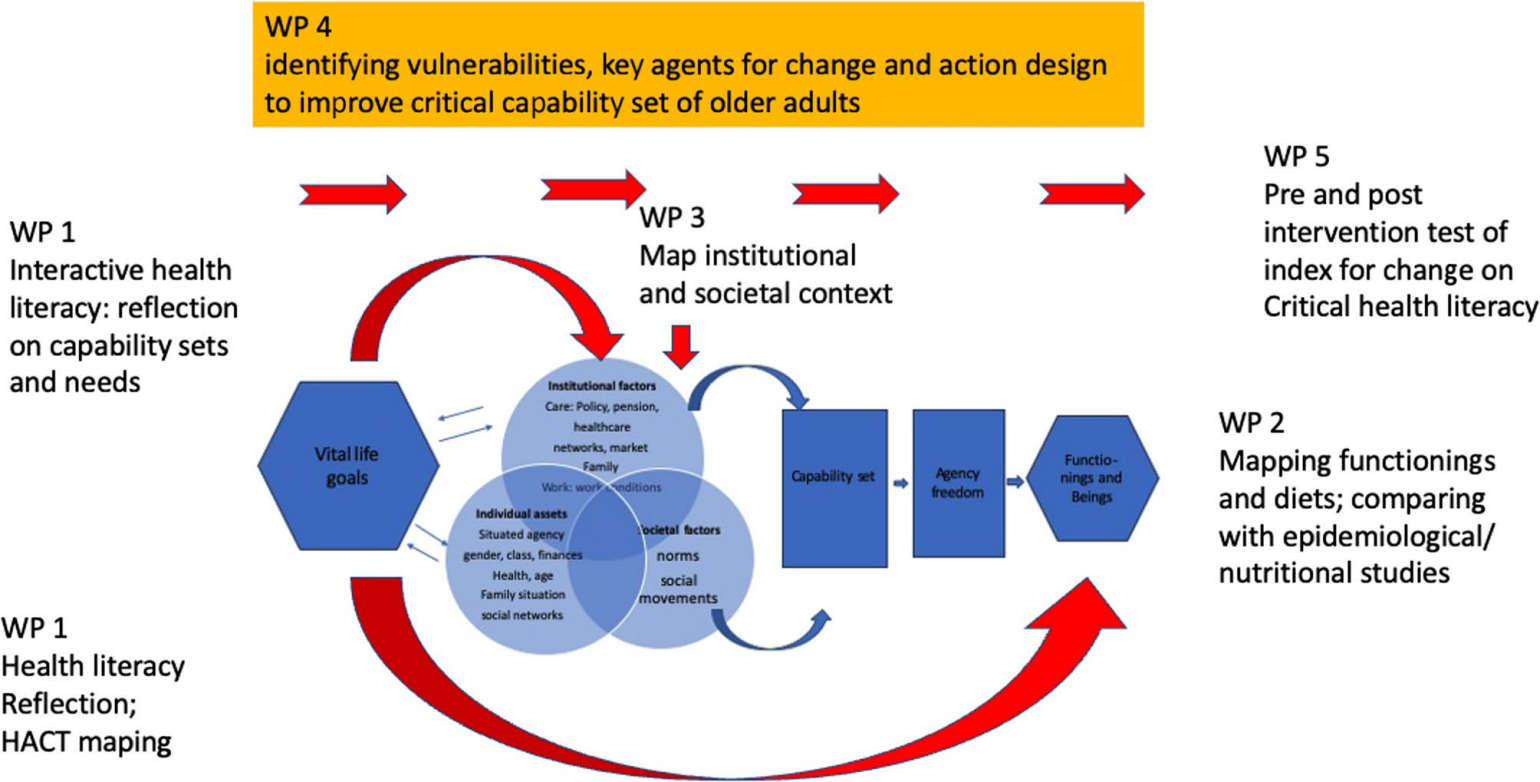
Participatory action research design based on capability approach

Thus, this study (see Fig. 2), against the backdrop of WTH/capability theory and a decolonial health literacy perspective, is to take departure from IOAs’ perception on health, i.e. their own life goals concerning healthy and good diet and judgements over how their desires are met and what their capability sets are to achieve this (WP1). In the second stage, the nutritional criteria of traditional food, desired food, and available food is explored with the help of nutritional expertise. Among others, register based health data (Sweden), alternatively newly conducted survey data (in India) is analysed (WP2). Following the mapping local care/food provision regime for OAs and institutional, normative barriers and resources for achieving healthy diets (WP3), key local stakeholders, including Indigenous agents and organisations, are involved in an innovative developmental project (WP4), on how OAs’ dietary needs and desires could be enhanced in order to improve a key component of their health standards.

The outcomes of these studies, beyond the realisation of a local innovative project, include the development of comparative indexes and methodological summaries reflecting on working with decolonial participatory approach (WP5). This also leads up to policy recommendations as to how food provision for IOAs could be developed in specific local/national contexts (WP7). The results are also to be disseminated through research articles (WP8).

### Locality

**Fig. 3:**
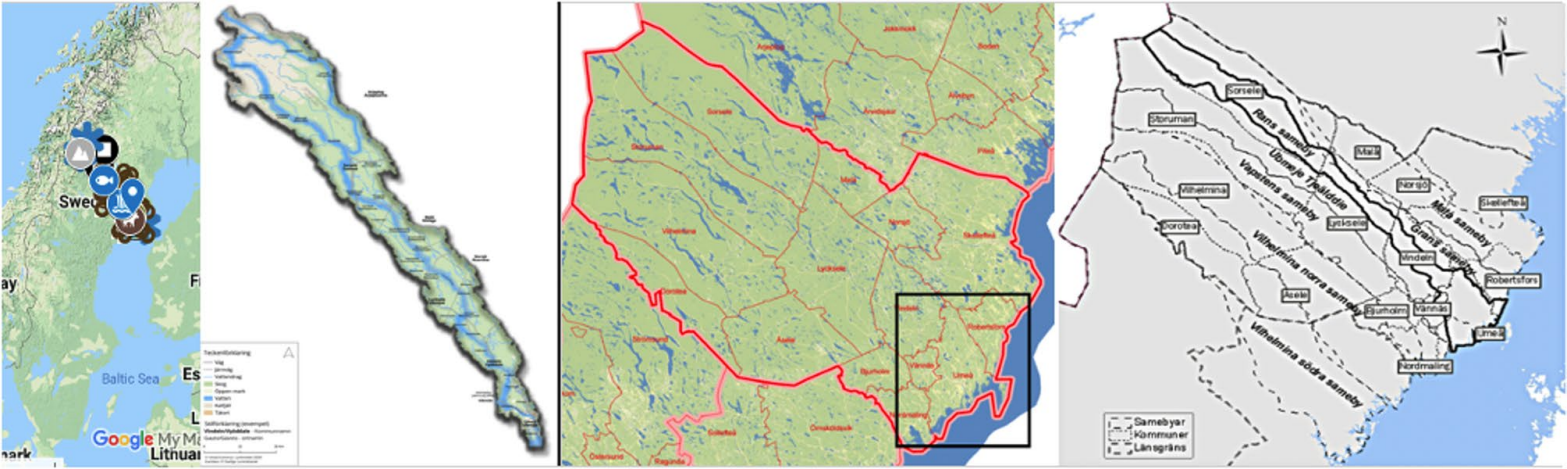
Figure on four different boarders: Vindelälven Juhtáhkka UNESCO area in Sweden, municipalities and regions, reindeer herding communities

In both countries, the selection of the site is chosen out of a dual criterion. For the one we are to identify socio- spatial units held together by a kind of environmental, ecological, social, economic and cultural cohesion, which is not necessarily the same as administrative units created by the state. In Sweden, the chosen unit is the Vindelälven Juhtáhkka biosphere reserve (see Fig. 3), a socio-ecological unit along the river Vindelälven [67]. Reindeer herders migrate between winter and summer grazing areas along this complex ecosystem of 1.3 million hectares, while they are semi-sedentarized, and have housing both along winter and summer areas. The more permanent of these is typically in a municipal area with good services, including school, health and old age services. According to the second criteria Umeå was chosen as a municipal centra, which provide for the care of the older Sami adults with *SCGM* status.

**Fig. 4:**
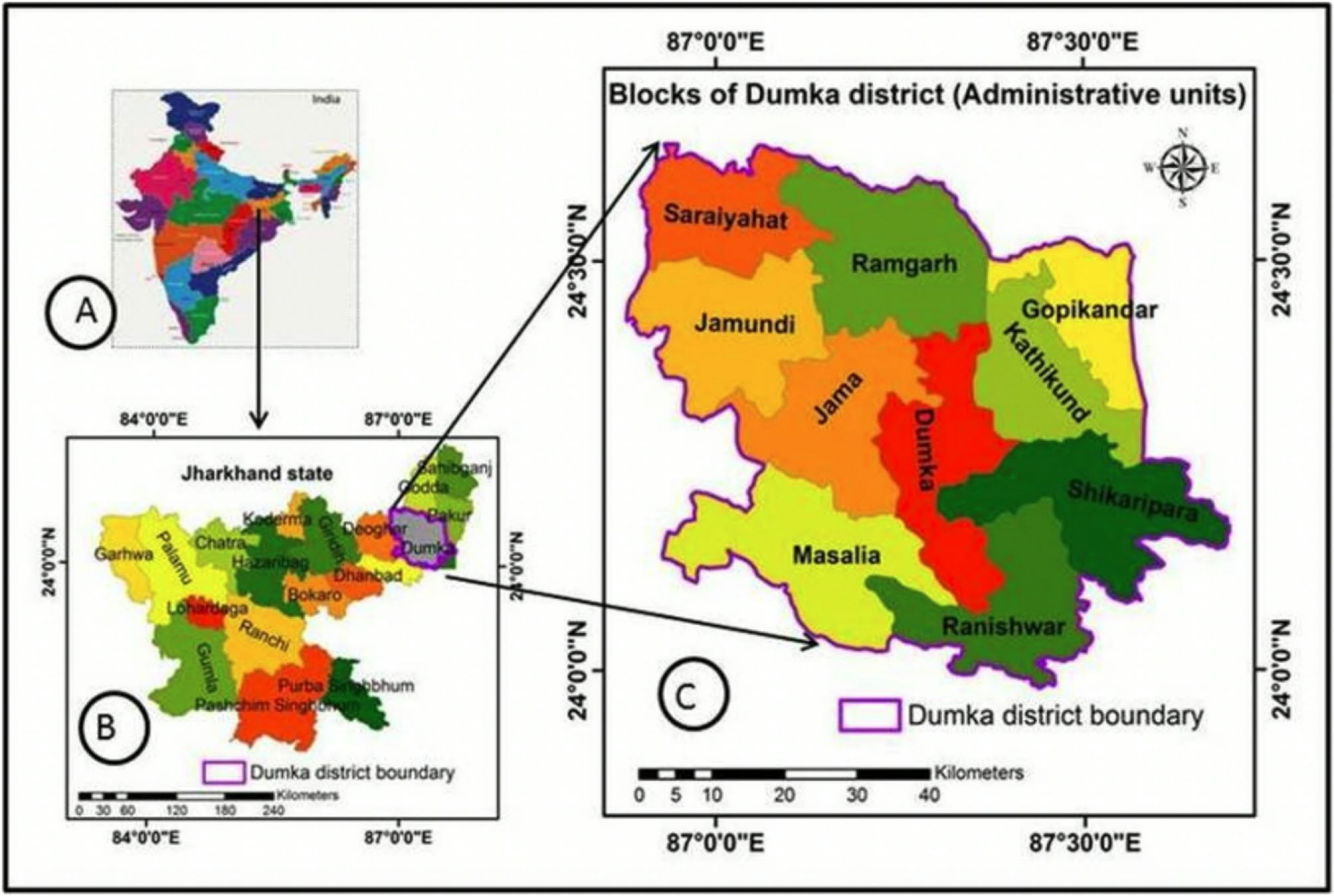
Dumka district in Jharkhand and its various administrative blocks (Ahmad and Goparaju)

In India the focus will be on the Mal Paharia tribe. Mal Paharias are mostly located in Dumka and nearby Pakur district of Jharkhand [23] (see Fig. 4). Their population in Dumka district is around 31,550 and is spread across 610 villages in the 10 blocks of the district. The study will be carried out in 20 selected villages of Kathikund and Dumka blocks (a block is defined as an administrative subdivision of a district) of Dumka district in Jharkhand, India.

### Criteria of inclusion and exclusion

Inclusion for study 1 + 3 are persons who are older than 65 years in Sweden. Within this group of IOA, those applying for care inputs from municipal organisations in Sweden are to be chosen for the participatory study. In India, the criteria need to be adjusted to IOA older than 55, due to the difference in life expectancy among Mal Paharia. Research persons should be capable to express free will. Inclusion criteria for study 3 is experience to work with authorities or organisations engaged with Indigenous people, Food security or OAs. Inclusion criteria for study 4 is the same as for study 1 and 3.

### WP 1 qualitative perspectives of IOAs food consumption

This research departs from the experiences and reflection of older Indigenous persons. This first study is also the point of departure for the following work packages, PAR for improved local provision of care and food for IOA.

#### Sampling

In Sweden 15–20 OAs of Sami origin are invited to participate through municipal care providers as well as through Sami organisations active within the municipality. In India 20–25 OAs of Mal Paharia origin through community health workers and village head will be identified for in depth semi-structured interviews in each of the 20 villages till saturation is attained.

*Transect walk and social mapping* will be carried out in the selected villages/localities to understand the landscape, and distribution of resources in food access, including market access for different foods and their prices, such as in India the local weekly market (*haat*) or reindeer meet through family networks in Sweden. OAs are invited to show and experience food memory places in two seasonal walk-abouts.

*Food-story interview* includes a life story [68] with semi-structured set-up coverning key life-cycle events and current living arrangements, conducted with OAs – with food as a central theme. Doing so, seasonal variation of food and origins of food are to be discussed, since the traditional food system adapts to seasonal variations in availability of food locally. Inspired by *WTH and capability theory* focusing on OA’s vital life goals OAs are asked how they would like to form their diet, if they can reach their goals, whether they have the resources (material and immaterial) to fulfil it, and whether whom they can ask for help to achieve it. This qualitative study is also of importance for identifying the key food items in the diet of OAs that can give indication to how previous survey data on health and food consumption of OAs can be used for further analysis. If deemed necessary, complementary interviews may be arranged with *family members* and informal care providers to explore care relations and food provision networks.

*Focus group discussions* FGD [69] will complement interviews. In India at least 4–5 OAs in each of the 20 villages until data saturation is achieved. The core FGD team will include experts in nutrition, anthropology and a trained interviewer. In India the core team is assisted by a village elder and/or the community health and nutrition worker of the village and trained local field workers fluent in the native Paharia dialect for facilitating the discussions. In India these will take place in the open areas of villages (COVID appropriate behaviour). In **Sweden**, a trained Sami chef Ann Sparrock is to lead the FGD team. FGDs follow up food memories concerning Indigenous Foods (IF) as well as discuss current desires and concerns with food and range of foods, including IFs, consumed. Freelisting of IF is followed by pairwise ranking of IF acc to centrality for FGD members. Questions concerning IOAs’ ability to access IF are raised to estimate their level of empowerment [70].

#### Analysis methods

Considering the varied components of methods in WP1, life story and food story interviews are explored in multiple ways, such as:

- A list of IF and its geographic, seasonal and historical timeline is constructed. Sami e.g. recognise eight seasons. Edible plants and their nutritive parts (leaves, roots…) are categorised. Methods of consumption and preparation categorised.
- Establishing OAs’ locally specific desired food preferences by pairwise ranking.
- Mapping of OAs’ material and immaterial resources, including informal networks to access food Accessibility matrix (markets, prices, in kind access, municipal service).
- A problem tree analysis is executed to explore the root causes and the possible solutions for food security and sovereignty for OAs.

### WP 2 quantitative study on nutritious index

The quantitative study will be performed differently in India and Sweden, with focus on food security in India and on food sovereignty in Sweden.

In Sweden data collected in 2021 through the population-based Sámi Health on Equal Terms Study (SámiHET) will be used. The SámiHET questionnaire was developed by a research team at the department of Epidemiology and global health at Umeå university (EpiGH) in collaboration with the Swedish Sámi Parliament and the Swedish National Public Health Agency. The SámiHET questionnaire was sent to a population of 9,260 persons that could be identified as Sami by a combination of indicators, such as people registered in the electoral registry of the Swedish Sámi parliament, the reindeer mark owner registry and reindeer herding entrepreneurs of the national registry on entrepreneurs. The questionnaire contained 81 questions on health, living conditions and culture. The questionnaire was sent to persons aged 18–84 years and 3,790 persons responded (40.9% response rate) of which 1124 persons (569 women and 555 men) were older than 65 years [71].

Five questions related to food patterns were included in the protocol. Four of them were standard questions from the Swedish Health on Equal Terms survey, mirroring consumption of vegetables, fruit and berries, fish and sweetened beverages. The fifth mirrored consumption of reindeer and elk meat, as an indicator of a traditional Sámi Food pattern. Variables on participation in general and Sami activities (e.g. participating in reindeer roundabouts) are relevant to active ageing.

The analysis of SámiHET data will identify the food indicators on consumption of IF and none IF food in different sub-groups of OA Sámi, based on geography, sustenance, language, and indicators of active ageing.

A possible extension, meaning access to more detailed Food Frequency Questionnaire data from elderly Sámi might be possible through collaboration with the HALDI project on Health in the Sámi and non-Sámi population in Jokkmokk or through collaboration with the MONICA project. The possibility of this will be explored.

**Fig. 5:**
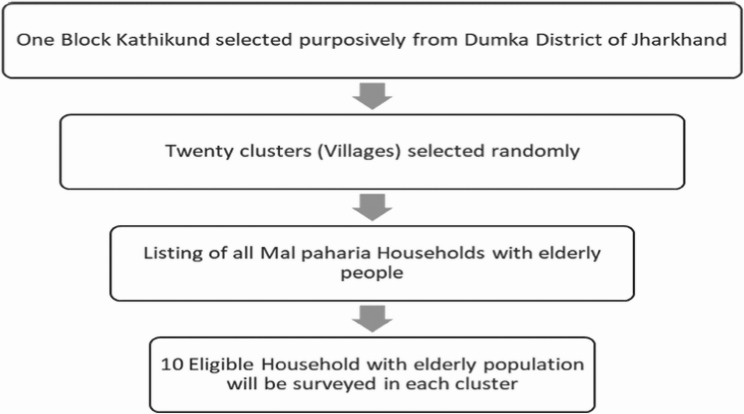
Two stage cluster random sampling strategy for selection of study subject

In India a two-stage cluster sampling will be done to enroll 200 elderly Mal Paharias (see Fig. 5).

The focus will be on food security from a nutrient analysis of diet consumed. This will be done by using a Food frequency questionnaire (FFQ) and a 24 h dietary recall method. Indian Food composition tables and other literature sources (Indian Council of Medical Research & National Nutrition Monitoring Board) will be searched to identify pre-existing information on their nutritive values. Food samples with missing or no available nutritive values in the secondary literature will be collected by the field team subject to their seasonal availability and sent to a NABL (National Accreditation Board for Testing and Calibration Laboratories) accredited laboratory for nutrient analysis.

A pre-tested FFQ will be used to elicit information on different types of Foods accessed by the study subjects. The 24-hour dietary recall method will be used to quantify the nutrient intake, which will be compared with dietary recommendations. Anthropometric measures will be taken to assess the state of nutrition in the elderly. An agriculture diversity tool and a market survey tool will be used to assess accessibility of food items for community in two crop cycles i.e., (i) summer and (ii) monsoon and/or winters. Information on type of food variety (Indigenous/non-Indigenous or hybrid) as well as their sources such as agricultural land by regular or traditional jhoom farming, forest land, kitchen gardens (baari), and water resources will be registered.

### WP 3 care regime study

WP3 connects to WP1, where the focus is on Indigenous adults and their informal caretakers. Following the principles of care and food regime analysis the specific features of the local care/food regimes, i.e. how state, civil society, market and family institutions interact in care/food provision for OAs, are unpacked.

#### Sampling

Stakeholders engaged with food provision for OAs are contacted top down (local/regional politicians, service providers) and bottom up (civil and Indigenous organisations).

In Sweden municipal as well as regional politicians, SCGM officials, regional authorities (County Board, SKR) administrative leaders for local care provision, care units employees engaged with food provision are contacted (top down) as local Sami organisation (Såhkie: Sami Cultural Association), close-by RHCs and national Sami organisations, including Slow Food Sapmi and SSR and authorities Sami Parliament.

In India one rural development block (Kathikund) is explored complementing WP1 by contacting decision makers for care provision, politicians, block, and district authorities engaged with Social Welfare, as well as local and district level Mal Paharia organisations (top down). Among Mal Paharia the village head is dahri assisted by godait and dewan [26]. Here those to be contacted include traditional Mal Paharia elders, the Panchayat, the Sahiyas (Accredited Social Health Activists), the Multi-Purpose Health Workers (Female), the Block Development Office, the District Collector, the Special Paharia Officer, the TPDS (targeted Public Distribution System) authority, the Medical Officer in Charge, the Forest Office, the Social Welfare Office. Furthermore, civil groups are contacted (bottom up), including the Paharias’ own, such as Gandhian and faith-based NGOs, Jan Swasth Abhiyan, Right to Food.

#### Research methods

This WP is primarily conducted by audio recorded qualitative semi-structured interviews, focus group discussions, participation in public and organisation-specific meetings, and observations. This case study is also preparatory for participatory innovation to be carried out in WP 4. For this purpose, workshops are arranged to discuss results from WP1 and 2 and create forum for OAs and Indigenous organisations to identify Indigenous food objectives for OAs as part of the capacity building principles.

#### Analysis

The analysis of the data will proceed along principles of social justice [15] making inquiries on the principles of participation, recognition and distribution concerning delivery and accessibility of food/resources to acquire food to IOAs in the given local food/care regime. How minority rights are respected in conduct with authorities, NGOs.

Aspects of capability theory [14, 55] are relevant in exploring the institutional and normative embedment of IOAs’ food-provision.

### WP 4 participatory implementation study

The two participatory implementation studies aim to improve food security and sovereignty for OAs in the chosen community by means of participatory action research, which is built up through the preceding project steps WP1-.

3. In this WP, using the knowledge gained from OAs about their dietary needs and care regimes of provision an action plan is made together with OAs and local indigenous organisations as to which issue is possible to pursue taking the time and resource frame of the project and the possible collaboration with local authorities.

In Sweden the method developed and applied in some municipalities by Sparrock [21](2021) is followed. This process is based on a double movement: The one is a bottom-up process, based on the empowerment of Sami OAs in care services. Based on what IOAs identify as dietary needs and what they identify as traditional Sami food, the project team, aligned with representatives of local Sami organisation (Sahkie) engages in a collaborative process with municipal care providers to incorporate Sami food items in the diet. The top-down movement engages municipal politicians, SCGM officals, municipal decision makers of care provision and care units, care and food provision staff. Both those planning and preparing the food and those serving the food need to be involved to enhance understanding of Sami food culture and its importance for OAs’ needs. The plan is to invite Umeå municipality to collaborate in the project by co-financing the worktime of its staff during participation. Previous trials by Sparrock led to introduction of key traditional dietary items with some regularity to the food offered to Sami OAs in municipal care provision, which has improved their self-appreciation and well-being. Nonetheless, so far, this method and its outcomes have not been evaluated in a systematic matter and been made public.

In India the primary objective is to achieve food security needs, and simultaneously attend food sovereignty goals. A double movement is applied even here: a ‘bottom up’ process (Through Geriatric clubs- termed ‘Bonphool’-Flower of the forest) and Van Bandhu Kalyan Yojana, renowned MalPharia activists and leaders from Mal Paharia elderly clubs (Bonphool) to enhance the empowerment of Mal Paharia OAs in the community and another, ‘top down’ process, by formation of District Geriatric Committee- termed ‘Bonmali’- Protector of the forest with District collectors as head of the committee and rest of the stakeholders like nodal officers for Targeted Public Distribution System (TDPS), Mahatma Gandhi National Rural Employment Act (MGNREGA), three general government pension schemes (Old age, Widow & Disability), Deendayal Antyodaya Yojana-National Rural Livelihoods Mission(DAY-NRLM), National Social Assistance Programme (NSAP) and Chief Ministers Special Food Security Scheme for Particularly Vulnerable Tribal Groups [72].

The project plans to catalyse the formation of clubs for the Mal Paharia elderly. The clubs could hold weekly discussions on the topics that the elderly Mal Paharia themselves prioritise. There are certain government schemes available that they have not been able to access sufficiently- both due to a lack of awareness and due to various physical, social, and administrative bottlenecks. The clubs would identify these, and it is hypothesised that they will be empowered to access some of these existing schemes. An identified leader from each Mal Paharia elderly club (Bonphool) will attend quarterly meetings of the District Geriatric Committee (Bonmali) to discuss and eliminate administrative bottlenecks to attain food security and sovereignty among Mal Paharias in the district.

Project members are to facilitate knowledge-exchange based on results from WP1-3, which systematise food security and sovereignty concerns and empower Indigenous activists to raise concerns.

### Ethical considerations

Since this research is conducted with Indigenous people, special decolonial research ethics are followed as of the ethical research guidelines of the Swedish Reindeer Herder Association’s [73] which emphasize the right to define the subject of research, participation on equal grounds, and withdrawal. Having IOAs as research subjects raise additional issues of vulnerability. In Sweden, in many ways the power to decide eligibility for care is vested in the hands of professional care providers. Our aim is to facilitate improvement and restorative justice [68, 74] without contributing to harm.

Following Swedish Research Council’s and ICMR’s ethical guidelines, we ask informed agreement in writing from all research persons assured even by vocal recording for participants in Sweden as well as India. The research persons are also going to be informed that participation is voluntary, that refusing participation would not mean any disadvantages and that any data collected would be handled confidentially observing GDPR requirements. No access would be granted to third party without authorisation. Further ethical aspects are considered based on the World Medical Association Declaration of Helsinki (2002) [75].

Ethical approval is sent to the regional ethical review authority (EPN) in Uppsala in alignment with (79 6 §, 2003:460). SLU is to be seen as research principal and therefore responsible that the research is carried out in a proper way in an ethical sense. Ethical approval was taken From Institute Ethical Committee at RIMS Ranchi in India as per ICMR Guideline (IECReg. No. -ECR/769/INST/JH/2015/RR-21).

Quantitative data from the SámiHET study will be handled according to the ethical approval already attached to the study protocol (The Swedish Ethical Review Authority, Dnr 2020–04803 and Ö 70-2020/3.1). In brief this means that the Lávvuo research group (https://www.umu.se/forskning/grupper/lavvuo/) were contracted by the Sámi parliament in Sweden and will continuously consult with its mandated members throughout the process.

## Discussion

### Contribution of international collaboration

India has a significant population of ST and government policies identified several as Particularly Vulnerable Tribal Groups. Mobilisation for the improvement of ST’s condition has been also motivated by understandings of the colonial past. There is a vibrant engagement both academic and via nation-wide civil organisations, such as SANMAT and AIILSG utilising PAR methods. Meanwhile, many in Sweden are unaware of Sweden’s colonisation of the North, large natural resource strong areas and how this still happens through displacement of Sami Indigenous practices. The Swedish welfare state secures OAs basic needs are met. However, the service passivizes OAs and is not reflexive on Indigenous older adults IOA traditions. IOAs bare both the knowhow of Indigenous practices and memories of postcolonial traumas. Drawing from transnational experiences of colonisation as well as mobilisation through civil movements transnational collaboration of academics can facilitate better understanding of IOAs situation in both contexts.

Transnational Collaboration projects across organizational and cultural boundaries extends the possibilities of discovery and provide findings beyond what one team could achieve alone. For rarely studied in-depth and longitudinally, the dynamics of Indigenous older adults and for better understanding of their food security and sovereignty issues- the perspectives of scientific team members from two such vastly different countries and cultures are crucial. Internationalization by transnational collaboration can promote sustainability surrounding their betterment in different ways: the transnational networks will provide channels to diffuse relevant knowledge and will be integral in facilitating processes of mutual learning. It will enhance intercultural sensitiveness and allow the pooling of often scarce resources despite of challenges like spatial distance and differences of culture, language.

### Transdisciplinary approach

Food is crucial for the health status of Indigenous older adults (IOA). How food is accessed by Indigenous older adults differs largely between India and Sweden, yet variation is great even within a given country [8] depending on the local care/food regime and the balance between key agents (municipal/state, family, civil and market) in terms of care/service provision and economic responsibility for care. The planned studies are to engage Indigenous older adults to account on what food means for them and how their capabilities are to obtain culturally and nutriently adequate food in a given regional unit. We explore how care provision is embedded in the local care regime and how this local care regime is part of a broader, regional, national system with the intention to improve current provision of food and through this the health of IOAs in a filed identified as central and feasible through a PAR process. Comparative synergies are expected to emerge, by this systematic unpacking of the interplay between these institutions of care and food provision. The study balances between structural, organizational analysis the study is to explore the importance of identity and culture for the health and resilience of Indigenous minorities and older persons within these minorities as well focus on health.

Thereof, this study is to be realised by a multidisciplinary team including social medicine, epidemiology, sociological and anthropological expertise, and all with diverse experience in the social gerontological field. However, beyond contributing to knowledge building within each of these fields, the aim is to bring knowledge work together and explore interdisciplinary and transdisciplinary advantages. IOAs food is explored from multiple academic lances: as depository of Indigenous knowhow of uncultivated food resources adequate to local conditions, as food memories connected to lifestyles and identities, and as current intake influencing health status, as relations between social conditions of poverty and malnutrition. These interdisciplinary synergies can also lead to transdisciplinary encounters exploring the health of IOA from a holistic perspective, rather than from the angle of particular disciplines.

Most importantly the study is based on decolonial approach which does not assumes an a priori hierarchy between traditional/Indigenous knowhow and instead builds on a collaborative knowledge production and interfaces between the two.

### Gender aspects

The study is expected to reveal comparative gendered (dis)advantages concerning food security and food sovereignty of IOAs in the two countries.

Gender inequalities of IOAs’ health are rooted in social division of resources and tasks that are embedded in the intergenerational relations of care and work [76]. Care is seen as an act of “love” guided by the need of those cared for [77]. It is in most societies an unpaid female task causing dependency on the male heads of family [78]. An imbalance prevails even in two-earner societies, like Sweden, where women’s labour force participation is high. Elderly women have lower pensions, and thereof lesser means to realize goals. Women continue with their caring, incl food preparation, responsibilities even in older age.

Sami women enjoyed a more equal position historically, which was pressed back by state regulation of reindeer herding favourable for male inheritance [79]. Fishing, gathering and reindeer herding were to varying degree shared activities, while hunting was men’s duty while women did most, yet not all food preparation. Food interviews could map how gendered task sharing is reflected in IF knowhow. In modern times Sami women obtain higher education compared to men [80] and by this often have enjoyed economic independency which could lead to independent economic security at old age and expectedly low gender gap in nutrition related health.

Older women’s dependency is especially profound in rural India, where women do not inherit and lack personal property making them easier offers of elderly abuse [34]. Since the expected institution to take care the old is the intergenerational family [81], dysfunction of support places elderly into vulnerability.

Social/pension security covers only 20% of the elderly population [82]. Not surprisingly, women in poverty show grater dissatisfaction with life [83]. Kshatriya & Achary [84] study for tribal groups of four regions of India confirms that the gender gap in the malnourishment for the disadvantage of women is maintained through life. Furthermore, the proportion of those malnourished increases with age.

## Data Availability

No datasets were generated or analysed during the current study.

## Abbreviations

OA: Older adults
IOA: Indigenous Older Adults
ST: Scheduled Tribes
PVTG: Particularly Vulnerable Tribal Groups
SCGM: Sami cultural governance municipality
